# *In vitro* metabolomic footprint of the *Echinococcus multilocularis* metacestode

**DOI:** 10.1038/s41598-019-56073-y

**Published:** 2019-12-19

**Authors:** Dominic Ritler, Reto Rufener, Jia V. Li, Urs Kämpfer, Joachim Müller, Claudia Bühr, Stefan Schürch, Britta Lundström-Stadelmann

**Affiliations:** 1 Institute of Parasitology, Department of Infectious Disease and Pathobiology, Vetsuisse Bern, University of Bern, Bern, Switzerland; 2 Division of Systems and Digestive Medicine, Department of Surgery & Cancer, Imperial College London, London, United Kingdom; 3 Department of Chemistry and Biochemistry, University of Bern, Bern, Switzerland

**Keywords:** Biochemistry, Metabolomics, Microbiology, Parasitology

## Abstract

Alveolar echinococcosis (AE) is a zoonotic disease that is deadly if left untreated. AE is caused by the larval metacestode stage of the cestode *Echinococcus multilocularis*. Better knowledge on the host-parasite interface could yield novel targets for improvement of the treatment against AE. We analyzed culture media incubated with *in vitro* grown *E. multilocularis* metacestodes by ^1^H nuclear magnetic resonance spectroscopy to identify the unknown metabolic footprint of the parasite. Moreover, we quantitatively analyzed all amino acids, acetate, glucose, lactate, and succinate in time-course experiments using liquid chromatography and enzymatic assays. The *E. multilocularis* metacestodes consumed glucose and, surprisingly, threonine and produced succinate, acetate, and alanine as major fermentation products. The metabolic composition of vesicle fluid (VF) from *in vitro* grown *E. multilocularis* metacestodes was different from parasite-incubated culture medium with respect to the abundance, but not the spectrum, of metabolites, and some metabolites, in particular amino acids, accumulated in the VF. Overall, this study presents the first characterization of the *in vitro* metabolic footprint of *E. multilocularis* metacestodes and VF composition, and it provides the basis for analyses of potentially targetable pathways for future drug development.

## Introduction

Platyhelminths (flatworms), including cestodes (tapeworms) and trematodes (flukes) cause a variety of human and animal infections. Currently, the highest ranked foodborne parasite in Europe is *Echinococcus multilocularis*, the small fox tapeworm^1^. Infection with this cestode causes the disease alveolar echinococcosis (AE) in humans. *E. multilocularis* is endemic to the Northern hemisphere including areas in Europe, Asia, and North America^2^. Over the recent years, *E. multilocularis* has been emerging in Europe, North America and Asia^3–5^. The life cycle of *E. multilocularis* includes canids as definitive hosts, and voles as intermediate hosts. Humans, and other mammals such as dogs, captive monkeys, and beavers, can be infected as accidental intermediate hosts by ingesting parasite eggs shed within the feces of definitive hosts. Thereby, these accidental hosts can acquire the disease AE. In humans, *E. multilocularis* grows as larval metacestodes, which primarily affect the liver and cause the disease AE, but they can also form metastases in other organs, especially at the late stage of infection^6^. Human AE is a chronic disease with extensive morbidity and mortality if remained untreated. Curative treatment is based on radical surgical resection in combination with temporal chemotherapy, applied in 20 to 50% of all cases^6^. Lifelong chemotherapeutical treatment based on benzimidazole-carbamate derivatives is applied if radical surgery is not possible. This treatment, however, may fail, and has a limited potential for complete cure. In addition, it can induce severe, life-threatening side-effects^7^. With increasing numbers of patients and no alternative to benzimidazoles developed so far, new and better treatment options are urgently needed^8^.

Morphologically, metacestodes are multivesicular larval stages surrounded by an acellular, carbohydrate-rich laminated layer^8^. Inside the vesicles, the parasite tissue is comprised of the cellular germinal layer, which covers the inner surface of the laminated layer. The germinal layer consists of muscle cells, nerve cells, glycogen storage cells, connective tissue, and undifferentiated stem cells^9,10^. The syncytial tegument, as the most outer part of the germinal layer, constitutes the interface between the germinal and the laminated layer, and it forms microtriches protruding from the germinal layer into the laminated layer^11^. Release of small vesicles from these microtriches has been described, which could be involved in release or uptake of metabolites^12^.

Metabolomics provides an excellent tool to characterize host-parasite interactions^13–15^. Metabolites such as amino acids, lipids, or sugars directly reflect the biochemical activity and the metabolic state of cells or tissues^16,17^. Various studies applied metabolic profiling to study diseases caused by flatworms *in vivo*, namely *Echinostoma caproni* in mice^18,19^, *Fasciola hepatica* in rats^20^, *Onchocerca volvulus* in humans^21^, *Opisthorchis felineus* in hamsters^22^ and humans^23^, and *Schistosoma mansoni* in mice^24–26^ and humans^27^. Only few studies directly assessed the metabolic composition of flatworms like *S. mansoni*^28^ and *F. hepatica*^29^. Cestodes were largely neglected in modern metabolomic studies, and no in-depth analyses have been performed to investigate the metabolic behavior of flatworms or the metabolic relationship between hosts and flatworms. Such metabolic information, however, could promote the development of therapeutic treatments, for example by blocking the nutrient uptake by the parasites. ^1^H Nuclear magnetic resonance (NMR) provides a, highly reproducible tool for untargeted footprinting of known and especially unknown metabolites with minimal interference due to sample preparation. Mass spectrometry (MS) is frequently used in metabolomics as well, and it is more sensitive than NMR. However, MS has a lower reproducibility and requires targeted pre-processing of samples, which narrows down the group of metabolites identified. Thus, NMR is the method of choice for untargeted metabolomic footprinting^30^. The metabolism of *E. multilocularis* is of particular interest, as genomic and transcriptomic analyses revealed that the parasite is deficient in large parts of amino acid, nucleotide and lipid synthesis, but has developed several protein families for nutrient uptake^31–33^.

*E. multilocularis* metacestode vesicles are filled with vesicle fluid (VF), which contains parasite and host components, and it is assumed to be involved in nutrition storage. The analogous hydatid fluid of *E. granulosus*, a close relative to *E. multilocularis*, contains pools of amino acids, sugars, lipids, fermentation end products such as acetate, alanine, lactate, and succinate, in metacestodes harvested from naturally infected intermediate hosts or human patients^34–39^, or from experimentally infected mice^40,41^. *In vitro* uptake of cholesterol into *E. granulosus* cysts has been demonstrated by the use of radiolabeled lipids^42^. One earlier study addressed changes in metabolite composition of *E. multilocularis* VF *in vitro* upon drug treatment, identifying four major fermentation end products, namely acetate, alanine, lactate, and succinate^43^. In another study, the metabolites of *E. multilocularis* cysts grown in treated versus non-treated animals were compared, and most pronounced differences were found in the levels of acetate, alanine, glycerolphosphatidylcholine, glycine, glycogen, and succinate^44^. Thus far, no metabolomic study has been performed applying the defined *E. multilocularis* metacestode *in vitro* culture system^45,46^, which allows for further molecular analyses of interesting pathways.

*E. multilocularis* protoscoleces use glucose as a major energy source, accumulate glycogen *in vitro*^47^ and express all enzymes of glycolysis, fermentation, and the tricarboxylic acid cycle^48^. Decreased glucose levels are detected in all organs of *E. multilocularis* infected jirds, and accordingly also reduced glycogen levels in the livers of these infected animals^49^. This further strengthens the importance of the energy source glucose also in an *in vivo* setting. The metabolic end products of glucose as an energy source of *E. multilocularis* protoscoleces are acetate, lactate, and succinate^47^. In contrast to protoscoleces, information on energy and intermediate metabolisms of the disease-causing stage of *E. multilocularis*, the metacestode, is scarce. Here, we present a study identifying the metabolites consumed and released by *E. multilocularis* metacestodes under anaerobic growth conditions using ^1^H-NMR for the identification, and liquid chromatography as well as enzymatic assays for the subsequent quantification of selected metabolites. Our study shows that metacestodes secrete acetate and succinate at the expense of glucose, and that threonine is the major amino acid consumed from the medium.

## Methods

If not stated otherwise, all chemicals and materials were purchased from Sigma-Aldrich (Buchs, Switzerland). Cell culture media and fetal bovine serum were from Bioswisstec (Schaffhausen, Switzerland). Statistical analyses were performed using the software package R (V 3.4.1). Figure plotting was done with the R package ggplot2 (V 3.0.0) and figure layouts were finalized using Adobe Illustrator CC (V 22.0.1).

### Experimental design

**Figure 1:**
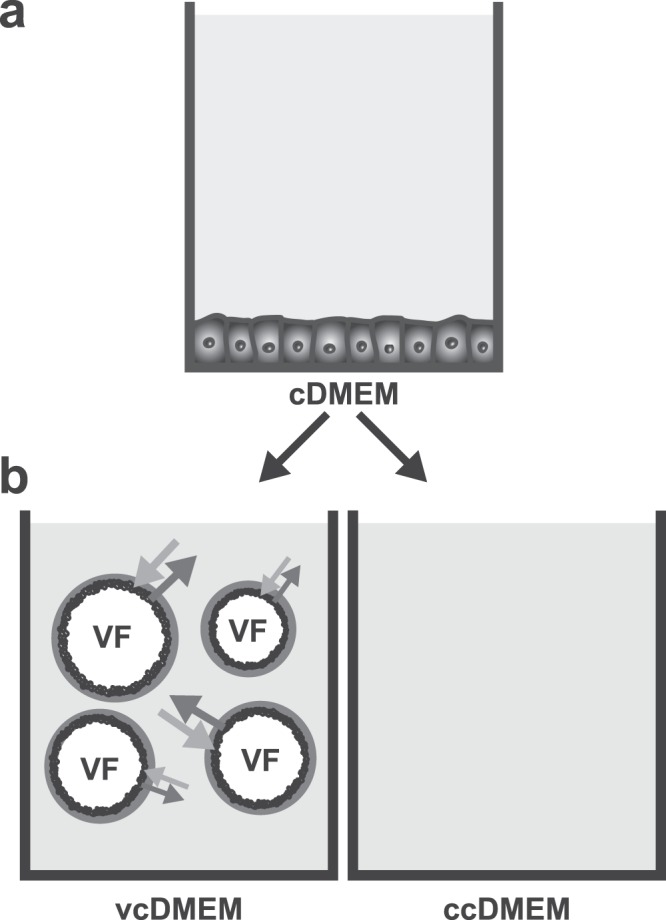
Graphical representation of the experimental setup. The experimental setup for the metabolic foot printing of E. multilocularis metacestodes *in vitro* included (**a**) medium preconditioning with rat hepatoma cells for four days in DMEM including 0.2% FCS to enrich the medium with host cell metabolites (cDMEM). (**b**) incubation of cDMEM with *in vitro* cultured E. multilocularis metacestode vesicles (vcDMEM) or mock incubation for control (ccDMEM) over 72 h (for ^1^H NMR analysis) or for the time-points 0, 2, 6, 10, 24, 48, 72 h (for quantitative measurements). During this time, metacestode vesicles consumed and released metabolites (indicated by arrows). Samples of vcDMEM, ccDMEM, and vesicle fluid (VF) were harvested for subsequent metabolite identification by ^1^H NMR, HPLC, or enzymatic assays.

To identify metabolic changes at the interface of the *E. multilocularis* metacestode with its growth medium, the *in vitro* setup depicted in Fig. 1 was applied. Reuber rat hepatoma cells (RH) were used to precondition culture medium for subsequent *E. multilocularis* metacestode vesicle incubation. This preconditioned medium was incubated *in vitro* with *E. multilocularis* metacestode vesicles. The metabolites in the corresponding samples of parasite-interacted medium (vcDMEM) and control incubated medium (ccDMEM) were analyzed by NMR spectroscopy. Metacestode vesicle fluid (VF) was also analyzed. To verify the NMR results and get a quantitative time-course measurement of selected metabolites, the same *in vitro* interaction setup was repeated independently, and samples analyzed by enzymatic acetate, glucose, lactate, and succinate measurements, as well as by free amino acid quantification with HPLC for different timepoints.

### *Echinococcus multilocularis* metacestode culture

*Echinococcus multilocularis* metacestodes vesicles (isolate H95) were cultured as described before^50^.

### Preparation of host cell conditioned medium

10^7^ RH feeder cells were seeded in a T175 cell culture bottle in 50 ml DMEM containing 10% FBS, 100 U/ml penicillin, 100 μg/ml streptomycin, and 5 μg/ml tetracycline, and incubated for one day at 37 °C, 5% CO_2_, humid atmosphere. Subsequently, RH cells were washed three times with PBS and 50 ml DMEM without phenol red. For the next four days, cells were incubated in 50 ml DMEM without phenol red and 0.2% heat inactivated FBS at 37 °C in 5% CO_2_, humid atmosphere. This host cell conditioned medium (cDMEM) was sterile filtered and directly used for incubation with *E. multilocularis* metacestode vesicles.

### Metabolite exchange between *E. multilocularis* metacestodes and host cell conditioned medium

To identify the metabolic footprint of *E. multilocularis* metacestodes, i.e. metabolites released or consumed by *E. multilocularis* metacestodes into or from the surrounding culture medium *in vitro*, the following setup was performed: Metacestodes were cultured for 12 weeks to reach a metacestode vesicle size of 2 to 4 mm in diameter. Metacestode vesicles were purified with 2% sucrose and PBS, and washed once in cDMEM. Subsequently, metacestode vesicles were incubated at a ratio of 1:2 in cDMEM for 72 h at 37 °C in a closed tube, resulting in the *E. multilocularis* metacestode vesicle interaction medium (vcDMEM). Integrity of metacestode vesicles was visually confirmed during the course of the experiment. Correspondingly, cDMEM only was incubated as control (ccDMEM) at 37 °C in a closed tube. For both media, ten biological replicates were analyzed. Medium supernatants were then stored at −80 °C for subsequent metabolite analysis. From the residual metacestodes, VF was extracted by washing the metacestodes 3 x in PBS and breaking them with a 1 ml pipette tip. Metacestodes were centrifuged at 9’000 × g for 20 min, 4 °C. The supernatant (VF) was centrifuged at 12’000 × g for 20 min, 4 °C and stored at −80 °C for subsequent metabolite analysis. VF was analyzed in five biological replicates.

### NMR sample preparation and data acquisition

Medium and VF samples from the above-described interaction were further prepared and analyzed by NMR at the Imperial College, London, UK. Details on sample preparation and data acquisition are found in Supplementary Methods 1.

### Metabolite identification

Metabolites were identified using Chenomx NMR Suite (V 8.2; May-01-2016 with Java 1.8.0_74 (x86)), Human Metabolite Database (HMDB)^51^, 2D NMR spectra and the published 2D NMR data^51,52^, and statistical total correlation spectroscopy (STOCSY) with the script IMPaCTS (v 1.0.0) in Matlab (V R2015b 8.6.0.267245)^53^.

### Data processing and statistical analysis of NMR spectra

Spectra were digitized into 20,000 data points with a resolution of 0.0005 ppm and peak regions between δ^1^H -0.01 to 0.01 for TSP and δ^1^H 4.68 to 5.04 for water were removed. Peaks were aligned using recursive segment-wise peak alignment (RSPA)^54^ and probabilistic quotient normalization was performed using IMPaCTS. To investigate changes in the relative concentrations of metabolites between the experimental groups, principal component analysis (PCA) and orthogonal projection to latent structure-discriminant analysis (OPLS-DA) were performed using the IMPaCTS script^55,56^. For OPLS-DA, the PLS and orthogonal components were calculated based on the mean-centered data scaled to unit variance. A seven-fold cross validation was used, and the corresponding cross-validation parameter was expressed as *Q*^2^*Y*. The total explained variation of the *X* matrix was indicated by the goodness of the fit (*R*^2^*X*). Figures were plotted as vector graphics in Matlab.

### Enzymatic assays for detection of acetate, glucose, lactate, and succinate

The levels of the metabolites acetate, glucose, lactate, and succinate were measured in the time-course samples (0, 2, 6, 10, 24, 48, 72 h) of vcDMEM and ccDMEM, as well as the endpoint sample VF (72 h) using the Acetate Colorimetric Assay Kit (MAK086, Sigma, USA), the Glucose-Glo TM Assay kit (J6021, Promega, USA), the Lactate-Glo TM Assay kit (J5021, Promega, USA), and the Succinic Acid Assay kit (K-SUCC, Megazyme, Germany) according to the manufacturers’ protocols. Measurements were made on an EnSpire 2300 plate reader (PerkinElmer Life Sciences, Schwerzenbach, Switzerland). Acetate, glucose, lactate, and succinate concentrations were calculated by linear regression based on the standard (0.00 to 10.00 nmol for acetate, 4.39 to 50.00 mM for glucose, 0.20 to 50.00 mM for lactate, and 0.13 µg to 4.00 µg total succinate) after subtraction of the respective enzyme blanks. Biological triplicates were analyzed for each sample and median and range (min, max) were calculated to represent the measured values. Linear reduction/accumulation rates were calculated using linear regression analysis in R and expressed as concentration changes in mM per hour.

### Amino acid quantification

To analyze the free amino acid content in the time-course samples (0, 2, 6, 10, 24, 48, 72 h) of vcDMEM and ccDMEM, the endpoint samples of VF (72 h), as well as germinal layer cell extracts of metacestodes at 72 h (for purification see Supplementary Methods 2), high-performance liquid chromatography (HPLC) was performed as described in Supplementary Methods 3. Biological triplicates were analyzed for each sample. For each amino acid and timepoint, median and range were calculated to represent the measured values. For metabolites with a linear change over time, reduction/accumulation rates were calculated using linear regression analysis in R and expressed as concentration changes in mM per hour. Other metabolites were analyzed by polynomial regression analysis in R and expressed as half-maximal or half-minimal times in hours.

Amino acid frequencies in proteins of germinal layer cells (cell preparation and hydrolysis in Supplementary Methods 2) were analyzed by HPLC accordingly. The amino acid content of metacestode proteins was calculated by subtracting the free amino acids from the hydrolyzed amino acids of the germinal layer cell extracts.

To analyze the relationship between the consumption and the concentration of essential amino acids in VF, germinal layer cell extracts, and metacestode proteins, linear regression and Cook’s distance outlier analysis were performed in R. Consumption of essential amino acids by metacestodes was defined as the difference between the pool sizes in ccDMEM and vcDMEM.

### Comparative statistical analyses of metabolite measurements

Integral intensities of the selected peaks from each metabolite identified by 1D ^1^H NMR were calculated for vcDMEM, ccDMEM and VF samples in Matlab (V R2015b 8.6.0.267245) and compared with the metabolite concentrations measured by enzymatic assays for acetate, glucose, lactate, and succinate and by free amino acid quantification using t-distribution of Person’s product-moment correlation in R. Tow sample t-test (two-tailed) was performed between metabolites of vcDMEM and VF as measured by free amino acid quantification and enzymatic assays. *P* values were Bonferroni adjusted with a significance level of *α* = 0.05.

To determine the percentage of glucose used for production of acetate, alanine, lactate, and succinate, pyruvate equivalents were calculated from the concentrations derived from enzymatic assays and free amino acid quantification. Thereby percentages were calculated by setting the glucose pyruvate equivalent concentration to 100%.

## Results

### The metabolic footprint of the *Echinococcus multilocularis* metacestode in culture medium

**Figure 2:**
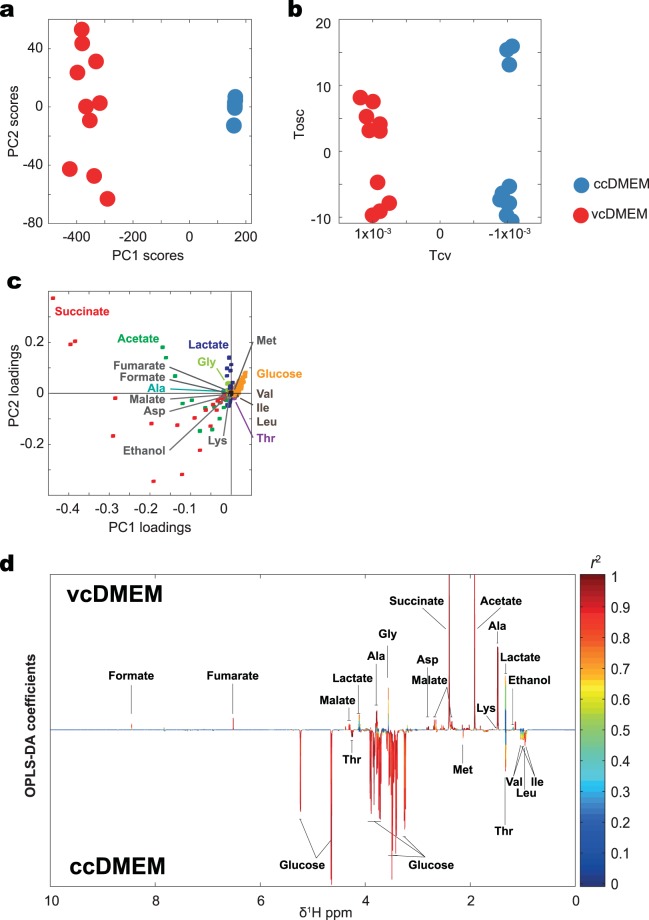
Multivariate statistical analysis of ^1^H NMR spectra. Multivariate comparison between vcDMEM (red, n = 10) and ccDMEM (blue, n = 10) acquired from 1D ^1^H NMR spectra after normalization. (**a**) Principal component analysis (PCA) scores plot of vcDMEM and ccDMEM. Groups are separated in the first principal component (PC1) with an explained variance of 98.39%. (**b**) Respective orthogonal projection to latent structure discriminant analysis (OPLS-DA) scores plot of vcDMEM and ccDMEM also clearly separated the two groups. Tcv depicts the inter-group variation, Tosc the intra-group variation. (**c**) Annotated PCA loadings plot of vcDMEM and ccDMEM. Annotated metabolites are indicated by various colors. (**d**) Annotated OPLS-DA coefficient plot of vcDMEM and ccDMEM. The x-axis depicts the chemical shifts in ppm. Peaks orientating upwards form the baseline show higher metabolite abundance in vcDMEM, from the baseline downwards pointing peaks show higher abundance in ccDMEM. The significance that each peak contributes to the difference between the two groups is indicated color-coded from blue low to red (scale on the right). The total explained variance (*R*^2^*X*) was 0.98 and the corresponding cross-validation (*Q*^2^*Y*) was 0.99. Amino acids are annotated using the three-letter code, for all other metabolites, full names are given.

Culture media containing *E. multilocularis* metacestode vesicles and control media (see Fig. 1) were analyzed using 1D ^1^H NMR spectroscopy to identify the metabolic footprint of *E. multilocularis* metacestodes *in vitro*. Representative 1D ^1^H NMR spectra are shown in Supplementary Fig. 1a,b. Multivariate statistical analyses including PCA and OPLS-DA were performed on the ^1^H NMR spectral data to investigate the metabolic differences between vcDMEM and ccDMEM. Clear group separations between vcDMEM and ccDMEM were observed in the PCA scores plot along the first principal component with an explained variation (*R*^2^*X*) of 98.39% (Fig. 2a). OPLS-DA scores plot also showed a clear separation between the two groups with a total explained variance of *R*^2^*X* = 0.98 and the corresponding cross validation value *Q*^2^*Y* = 0.99, which indicates a high goodness of fit (*R*^2^*X*) and predictability (*Q*^2^*Y*) (Fig. 2b). Thus, substantial differences between vcDMEM and ccDMEM were detectable by ^1^H NMR, reflecting the parasite-induced changes in the culture media.

Upon metabolite annotation of the ^1^H NMR spectra of vcDMEM and ccDMEM, a total of 21 metabolites were identified (Table 1, Supplementary Fig. 1a,b). To identify differences in the metabolite levels between vcDMEM and ccDMEM, the PCA loadings plot and the OPLS-DA coefficient loadings plot were derived from 1D ^1^H NMR spectra. The PCA loadings plot indicates that acetate, glucose, and succinate contribute most to the variation between vcDMEM and ccDMEM (Fig. 2c). Metabolites with a negative OPLS-DA discrimination score (Fig. 2d, Table 2), and thereby present at higher levels in ccDMEM than vcDMEM, i.e. being consumed by the parasite, were glucose, isoleucine, leucine, methionine, phenylalanine, threonine, tryptophan, tyrosine, and valine. Accordingly, several metabolites were released by the parasite with a positive OPLS-DA correlation coefficient (Fig. 2d, Table 2), exhibiting higher levels in vcDMEM than ccDMEM: acetate, acetone, alanine, aspartate, ethanol, formate, fumarate, glycine, lactate, lysine, malate, and succinate. Note that acetone, ethanol, lysine, phenylalanine, tryptophan, and tyrosine showed only a low correlation coefficient.

**Table 1:** Identified Metabolites in ^1^H NMR spectra of vcDMEM, ccDMEM, and VF.

Metabolite group	Metabolite name	Moiety	Chemical shift [ppm] (multiplicity*)
Amino acids	Alanine	β-CH3; α-CH	1.48 (d); 3.79 (q)
	Aspartate	β-CH2(ii); β-CH2;	2.67 (dd) 2.82 (dd); 3.89 (dd)
	Glycine	α-CH	3.57 (s)
	Isoleucine	terminal-CH3/d-CH3; β’-CH3; γ-CH2; γ-CH2; β-CH2; α-CH	0.93 (t); 0.99 (d); 1.26 (m); 1.46 (m); 1.98 (m); 3.68 (d)
	Leucine	δ-CH3; δ′-CH3; β-CH2 + γ-CH; α-CH	0.96 (d); 0.97 (d); 1.73 (m); 3.73 (m)
	Lysine	ε-CH2	3.03 (t)
	Methionine	β-CH’; δ-CH3; β-CH; γ-CH2	2.12 (m); 2.14 (s); 2.20 (m); 2.65 (t);
	Phenylalanine	β-CH2’(ii); β-CH2(i); α-CH; 3,5-CH; 2,6-CH; H3	3.13 (dd); 3.29 (dd); 4.00 (dd): 7.34 (m); 7.40 (m); 7.44 (m)
	Threonine	γ-CH3; α-CH; β-CH	1.33 (d); 3.59 (d); 4.26 (m)
	Tryptophan	β-CH2(ii), CH, H5, H5, C2, C7, C4	3.31 (dd), 4.07 (dd), 7.21 (t), 7.29 (t), 7.33 (s), 7.55 (d), 7.74 (d)
	Tyrosine	β-CH2’; β-CH; CH; 3,5-CH; 2.6-CH	3.06 (dd); 3.20 (dd); 3.95 (dd); 6.91 (m); 7.20 (m)
	Valine	γ-CH3; γ′-CH3; β-CH2; α-CH	0.99 (d); 1.04 (d); 2.28 (m); 3.62 (d)
Organic acids	Acetate	CH3	1.93 (s)
	Formate	CH	8.46 (s)
	Fumarate	CH	6.52 (s)
	Lactate	β-CH3; α-CH	1.33 (d); 4.12 (q)
	Malate	β‘-CH; CH2(ii); α-CH	2.38 (dd); 2.68 (dd); 4.31 (dd)
	Succinate	2xCH2	2.40 (s)
Sugars	Glucose	β-H2; NA; β-H3; α-H2; α-H3; αH5 αH6 αH6; αH5 αH6 αH6; βH6; β-H1; α-H1	3.25 (dd); 3.47 (m); 3.50 (t); 3.54 (dd); 3.72 (t); 3.78 (dd); 3.84 (c); 3.90 (dd); 4.65 (d); 5.24 (dd);
	Myo-inositol	CH; 2xCH; 2xCH; CH	3.2912 5CH (t); 3.5458 (dd); 3.6306 (t); 4.0756 (t)
Organic compounds	Acetone	CH3	2.24 (s)
	Ethanol	CH3; -CH2	1.19 (t); 3.67 (q)

The table shows metabolite group, metabolite name, moiety, and the chemical shift in parts per million (ppm) including the multiplicity of identified metabolites for vcDMEM (n = 10), ccDMEM (n = 10), and VF (n = 5).

*s, singlet; d, doublets; t, triplets; m, multiplets; q, quartet; dd, double doublet.

**Table 2:** The metabolic footprint of *E. multilocularis* metacestodes *in vitro*.

Metabolite group	Metabolite name	OPLS-DA discrimination score (correlation coefficient)	Median integral (MAD) for vcDMEM	Median integral (MAD) for ccDMEM	fold change of released metabolites	fold change of consumed metabolites
Amino acids	Alanine	2.61 (0.99)	215.14 (±15.53)	35.34 (±0.31)*	6.09	
	Aspartate	0.11 (0.99)	15.67 (±0.61)	2.97 (±0.08)*	5.28	
	Glycine	1.46 (0.97)	88.22 (±4.60)	59.62 (±0.51)	1.48	
	Isoleucine	−0.25 (−0.81)	71.36 (±0.76)	83.76 (±0.82)		1.17
	Leucine	−0.47 (−0.96)	110.53 (±1.61)	148.34 (±1.22)		1.34
	Lysine	0.01 (0.11)	79.40 (±2.23)	78.72 (±0.64)	1.01	
	Methionine	−0.24 (−0.89)	16.76 (±0.18)	21.95 (±0.27)		1.31
	Phenylalanine	−0.001* (−0.01)	31.74 (±0.50)	32.37 (±0.31)		1.02
	Threonine	−0.21 (−0.99)	3.99 (±0.22)*	38.80 (±0.37)		9.72
	Tryptophan	−0.001 (−0.43)	3.08 (±0.05)	3.25 (±0.06)		1.06
	Tyrosine	−0.01 (−0.26)	32.32 (±0.30)	33.52 (±0.29)		1.04
	Valine	−0.28 (−0.81)	89.44 (±0.97)	103.68 (±0.87)		1.16
Organic acids	Acetate	23.00 (0.99)	691.00 (±36.56)	91.54 (±1.04)	7.55	
	Formate	0.16 (0.89)	10.32 (±0.75)	6.21 (±0.08)	1.66	
	Fumarate	0.37 (0.96)	9.78 (±1.10)	0.29 (±0.05)*	33.72	
	Lactate	0.32 (0.71)	208.80 (±4.08)	191.41 (±2.26)	1.09	
	Malate	0.29 (0.96)	23.27 (±3.46)	4.57 (±0.07)*	5.09	
	Succinate	59.89 (0.99)	1664.01 (±93.71)	4.74 (±0.14)*	351.06	
Sugars	Glucose	−3.28 (−0.98)	1430.80 (±111.80)	2790.20 (±24.25)	1.95	
Organic compounds	Acetone	0.01 (0.74)	4.31 (±0.13)	3.98 (±0.10)	1.08	
	Ethanol	0.06 (0.31)	58.76 (±1.12)	53.12 (±0.69)	1.11	

Given are metabolite groups, metabolite names, explained differences between vcDMEM/ccDMEM by OPLS-DA (OPLS-DA discrimination score), median integrals of metabolite levels in vcDMEM (n = 10) and ccDMEM (n = 10) with the median absolute deviation (MAD) in parentheses, and fold changes for released and consumed metabolites. Asterisks indicate not detectable metabolite peaks (baseline levels are given).

The amino acid threonine was found exclusively in ccDMEM, and only baseline levels were found in vcDMEM (Table 2). The amino acids alanine and aspartate, and the dicarboxylic acids fumarate, malate, and succinate were identified in vcDMEM with baseline levels in ccDMEM (Table 2). All other identified metabolites (acetate, acetone, ethanol, formate, glucose, glycine, isoleucine, lactate, leucine, lysine, methionine, phenylalanine, tryptophan, tyrosine, and valine) were found in both vcDMEM and ccDMEM (Table 2).

### Quantification of selected metabolites in culture media

**Figure 3:**
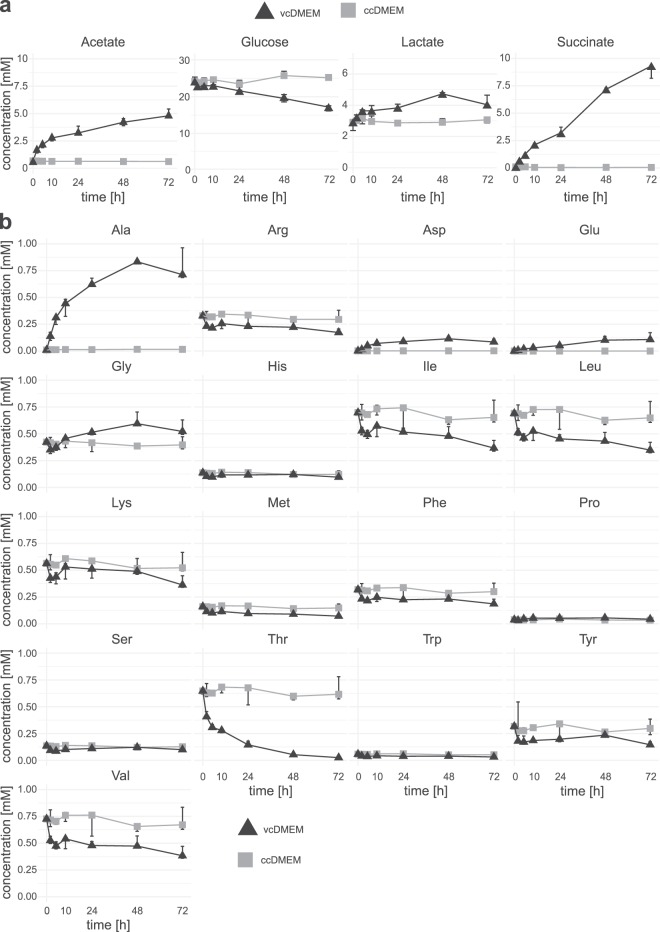
Quantitative time-course measurements of acetate, glucose, lactate, succinate, and amino acids in vcDMEM and ccDMEM. Metabolite concentrations were determined by (**a**) enzymatic assays (acetate, glucose, lactate, and succinate) and (**b**) free amino acid quantification by HPLC. Biological triplicates were assessed for each timepoint, and median concentrations and ranges are given for each metabolite for the timepoints 0, 2, 6, 10, 24, 48, and 72 h for vcDMEM and ccDMEM.

To confirm the ^1^H-NMR results and to quantify selected metabolites, concentrations of acetate, glucose, lactate, and succinate, as well as all amino acids, were determined by enzymatic assays or HPLC. Moreover, the changes in metabolite composition were analyzed in time-course experiments. Hereby, five amino acids not detectable by NMR were identified, namely arginine, glutamate, histidine, proline and serine (Fig. 3). The correlation of the concentrations determined by ^1^H-NMR and by quantitative methods were highly significant for both the samples of ccDMEM (Pearson: *r* = 0.99, *p* = 2.20 * 10^−16^) and of vcDMEM (Pearson: *r* = 0.91, *p* = 2.84 * 10^−6^). Amongst all quantitatively measured metabolites, glucose showed the largest concentration reduction after 72 h of culture with a stable median concentration of 24.71 mM in ccDMEM and a linear reduction to 16.73 mM in vcDMEM (linear reduction rate = 0.10 mM glucose/hour, Fig. 3). Acetate had a stable median concentration of 0.64 mM in ccDMEM. In vcDMEM acetate accumulated over the first 10 h with a linear rate of 0.23 mM acetate/hour. Between 10 and 72 h of *in vitro* culture, this rate dropped to 0.03 mM acetate/hour (Fig. 3). Lactate was at a concentration of 3.01 mM in ccDMEM and 4.04 mM in vcDMEM, with an increase mostly during the first 10 h (Fig. 3). Succinate showed a stable median concentration of 0.19 mM in ccDMEM that increased to 9.01 mM in vcDMEM (linear accumulation rate = 0.13 mM/hour). Alanine was stable at 0.02 mM in ccDMEM, but reached 0.71 mM in vcDMEM, plateauing after 48 h (half-maximal concentration reached after 13.46 h). Aspartate, glutamate, and glycine concentrations increased slightly over time in vcDMEM (Fig. 3). Conversely, the median concentrations of arginine, isoleucine, leucine, lysine, methionine, phenylalanine, threonine, tyrosine, and valine were decreased in vcDMEM after 72 h of incubation (Fig. 3). In particular threonine concentrations were rapidly diminished within 72 h of *in vitro* culture (half time = 9.23 h). The concentrations of histidine, proline, serine, and tryptophan did not change substantially between ccDMEM and vcDMEM during the incubation time (Fig. 3).

### Metabolomic comparison between vesicle fluid and culture medium incubated with *E. multilocularis* metacestodes

**Figure 4:**
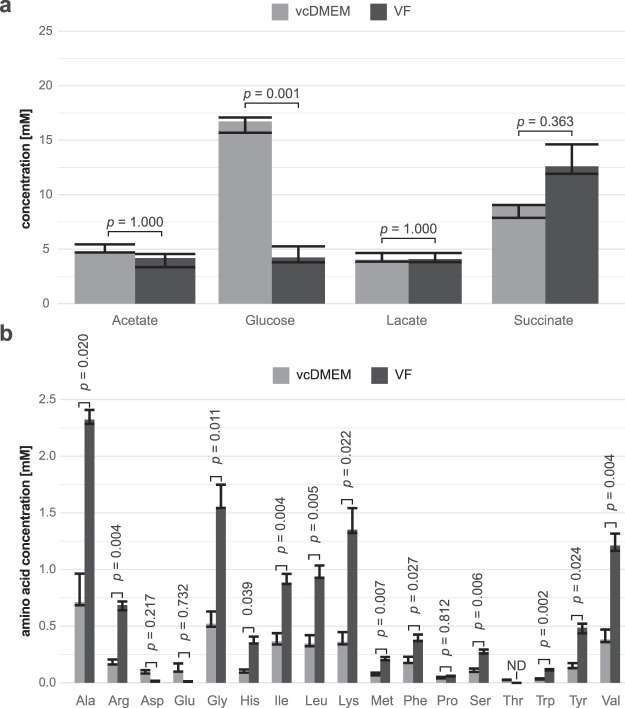
Concentrations of amino acids and selected metabolites in vcDMEM and VF. (**a**) Quantitative enzymatic assays of acetate, glucose, lactate, and succinate and (**b**) free amino acid quantification by HPLC were applied to measure metabolite concentrations in the samples vcDMEM and VF. Metabolite concentrations from three biological replicates are depicted as median and range in mM for the 72 h timepoint. The numbers above each bar pair represent Bonferroni adjusted *p* values derived from two sample two-sided T-test with a significance level of *α* = 0.05. ND = not detected.

In addition to the culture medium, we analyzed the pool sizes of metabolites in VF by ^1^H-NMR and by quantitative methods. A representative 1D ^1^H NMR spectrum for VF and analysis of metabolite abundance (peak intensities) is given in Supplementary Fig. 1c and Supplementary Table 1. A priori, the ^1^H-NMR-based metabolite profiles cannot be used for a quantitative comparison between culture media and VF, as they constitute different biofluids with different TSP values. Nevertheless, as for the culture medium, the correlation between the peak integral values obtained by ^1^H-NMR and by the values obtained by quantitative measurements (Fig. 4) was highly significant (Pearson: *r = *0.97, *p* = 2.92 * 10^−9^). Glucose showed significantly lower concentrations in VF than in vcDMEM and threonine was not detectable at all in VF. Conversely, alanine, arginine, glycine, histidine, isoleucine, leucine, lysine, methionine, phenylalanine, serine, tryptophan, tyrosine, and valine showed higher concentrations in VF than in vcDMEM. Aspartate, glutamate, lactate, proline, and succinate did not significantly differ between vcDMEM and VF (Fig. 4**)**. This difference in metabolite abundance and profiles between vcDMEM and VF indicates for selective metabolite transport through the metacestode wall.

### Amino acid composition of metacestodes: Is threonine overrepresented in vesicle fluid, in germinal layer cells, or in germinal layer proteins?

**Figure 5:**
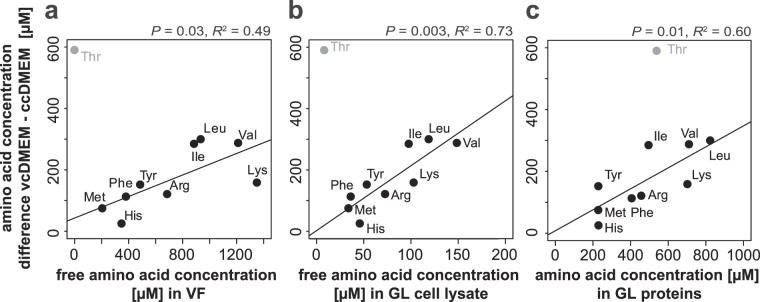
Free amino acid concentrations in vesicle fluid, in germinal layer cells, and germinal layer cell proteins. Amino acid uptake by *E. multilocularis* metacestodes (y-axis) was identified by the difference in amino acid concentrations in vcDMEM (n = 3) and ccDMEM (n = 3) as measured by HPLC. Amino acid consumption is depicted in relation to (**a**) free amino acids in VF (n = 3, x-axis), (**b**) free amino acids in germinal layer cells (n = 3, x-axis), and (**c**) amino acid composition of germinal layer cell proteins (n = 3, x-axis) as analyzed by HPLC. Median values in mM are depicted. The respective linear regressions are given excluding threonine. P values were determined by F-test.

The fact that threonine as the only amino acid showed an unexpectedly high difference between ccDMEM and vcDMEM prompted us to determine the concentrations of free amino acids in VF and germinal layer cells, as well as the amino acid composition of the proteome of germinal layer cells (Supplementary Fig. 2). We compared the concentration differences for the amino acids with lower contents in vcDMEM than in ccDMEM, i.e. consumed metabolites, with their corresponding concentrations in the samples VF, germinal layer cells and germinal layer proteome. All three regressions were significant, when threonine – identified by Cook’s distance outlier analysis - was removed (Fig. 5). Taken together, this confirms that the high threonine consumption by *E. multilocularis* metacestodes can neither be explained by protein synthesis, nor by storage in germinal layer cells or VF.

## Discussion

**Figure 6:**
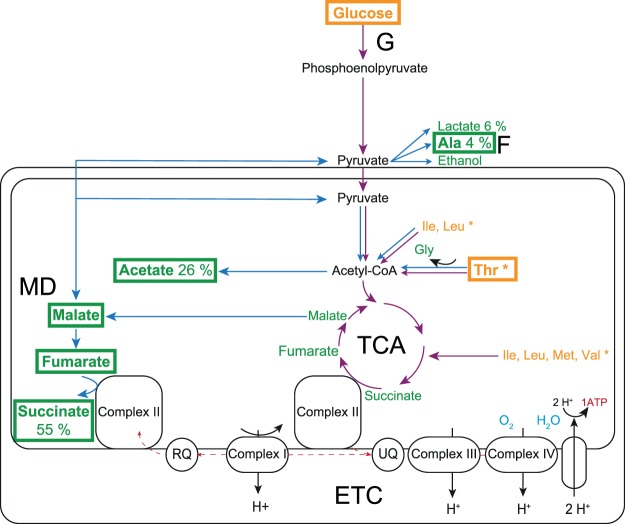
Overview of the mitochondrial energy metabolism and released and consumed metabolites by *E. multilocularis* metacestodes *in vitro*. The mitochondrial energy metabolism of *E. multilocularis* is given based on information from previous studies^32,33,47,48,62,75^. Aerobic pathways are given in purple, anaerobic ones in blue. Outside the mitochondrial membrane (black lines), glycolysis (G) and fermentation pathways (F, alanine, ethanol, and lactic) are depicted. Inside the mitochondrial membrane, malate dismutation (MD), rhodoquinone (RQ), tricarboxylic acid cycle (TCA), and ubiquinone (UQ) are given. Metabolites consumed *in vitro* by *E. multilocularis* metacestodes, as found within this study, are depicted in yellow, released ones in green. Metabolites that were released more than five times are indicated with bold letters and a rectangle (acetate, alanine, fumarate, malate, succinate; aspartate is not depicted). The pathways for consumed metabolites need to be further investigated. Putative pathways for use in the energy metabolism were determined in KEGG (indicated by asterisks)^76^. The most strongly consumed metabolites are indicated with bold letters and a rectangle (glucose and threonine). Percentages of these metabolites as produced from consumed glucose equivalents are given.

In the present study, we analyzed the metabolic footprint and requirements of *in vitro* cultured *E. multilocularis* metacestodes by ^1^H-NMR, by HPLC and by enzymatic assays to identify essential pathways that may constitute druggable targets. Overall, the results obtained within these independent setups and with three analytical methods were in good agreement. Confirming previously published results on protoscoleces^48^, glucose was the metabolite with the highest consumption, and most likely serves as the major energy source for both, metacestodes and protoscoleces. Under anaerobic conditions, the energy metabolism of *E. multilocularis* metacestodes is fermentative yielding acetate, alanine, lactate, and succinate as major end products, which accumulate in the culture medium. Glucose was metabolized yielding acetate (26%), alanine (4%), lactate (6%), and succinate (55%, see Fig. 6) as end products. The residual 8% of glucose equivalents were not traceable. The generation of succinate and acetate – in our study the most abundant end products – is of particular interest. Under anaerobic conditions, succinate and acetate are produced in mitochondria by an additional pathway, the malate dismutation pathway. Malate dismutation is found in helminths, marine invertebrates, and euglenids^57–59^. This pathway can be considered as a partial inversion or a shunt of the citric acid cycle. All knowledge on this pathway in helminths is based on nematodes (*Ascaris suum* and filaria), trematodes (*Schistosoma mansoni* and *Fasciola hepatica*) as well as the cestode *Hymenolepis diminuta*^58^. Under anaerobic conditions, pyruvate is imported into the mitochondria, but not decarboxylated via pyruvate dehydrogenase. Rather, it is carboxylated to oxaloacetate via pyruvate carboxylase. The reduction of oxaloacetate to malate, the conversion of malate to fumarate and the reduction of fumarate to succinate is performed by the corresponding enzymes of the citrate cycle working in the reverse direction, thereby regenerating NAD from NADH. The reduction of fumarate to succinate is dependent on complexes I and II of the mitochondrial respiratory chain. Complex I transfers electrons from NADH to rhodoquinone, an electron carrier with a much lower redox potential than the mammalian carrier ubiquinone. Rhodoquinone transfers these electrons to complex II which operates in the opposite direction than in oxidative phosphorylation, namely as a fumarate reductase instead of succinate dehydrogenase^58,59^. Succinate as a final product of this fermentative pathway is then excreted to the medium (see Fig. 6 for an overview). Some parasitic helminths further metabolize succinate to propionate or volatile fatty acids. So far, there is no indication that these reactions also take place in *Echinococcus*, as these metabolites have never been detected in the parasite, nor are the responsible enzymes identified in the *Echinococcus* genomes. Thus, for helminths, malate dismutation is an important pathway to eliminate redox equivalents under anaerobic conditions. By generating a proton gradient via complex I, it may even contribute to mitochondrial ATP synthesis^59,60^. To date, *Echinococcus* species were largely excluded from studies of the malate dismutation pathway, even though accumulation of succinate was described early, mostly in *E. granulosus* protoscoleces^47,61,62^ and cysts^37^, but also in *E. multilocularis* protoscoleces^47^ and metacestodes^63^. Absent in mammalian cells, malate dismutation may constitute a highly interesting drug target, which has been addressed in one study that investigated this pathway in protoscoleces of *E. multilocularis*^64^. Moreover, the high release of metabolic end products could modulate the host response, as it was shown that succinate and fumarate can induce host cell apoptosis^65^, and succinate stimulates IL-1β secretion in T-cells and could therefore cause inflammation^66^.

The second most abundant fermentation product of metacestodes detected in this study is acetate which confirms the results described for *E. multilocularis* protoscoleces^47^. The generation of acetate as fermentative end product may be a consequence of the malate dismutation pathway which shunts the citrate cycle (see above). Lacking oxaloacetate as acceptor, acetyl-CoA resulting from imported pyruvate or from lipid degradation^67,68^ transiently accumulates and is hydrolyzed to acetate, thereby regenerating coenzyme A. Acetate is then secreted.

The third major fermentation end product, alanine, is generated from pyruvate by transamination in the cytosol. Conversely, pool sizes of lactate, the classical fermentation end product in mammalian cells, do not markedly differ between the three fluids investigated in this study.

The difference in abundance, but not spectrum, of metabolites between vcDMEM and VF could be explained by the fact that small metabolites (alanine, arginine, glycine, histidine, isoleucine, leucine, lysine, methionine, phenylalanine, serine, tryptophan, tyrosine, and valine) pass the metacestode wall but are selectively accumulated inside. Myo-inositol was detected in traces in VF, not in vcDMEM, but no further focus was laid on this metabolite in the present study. Overall, our results on VF composition are in agreement with previous studies on the composition of *E. granulosus ex vivo* hydatid fluid^34,37,38,41^. The only difference is that in *E. multilocularis* metacestode VF, alanine is the most abundant amino acid whereas in hydatid fluid, glycine is the most abundant amino acid^34,41,69^. *E. multilocularis* releases traces of aspartate, glutamate, and glycine into the medium. Thus, we consider that in addition to alanine, these three amino acids can be synthesized by *E. multilocularis*. Based on transcriptomic information, the synthesis pathways for all other amino acids are incomplete or lacking^32^. Therefore, they have to be taken up from the culture medium. Most striking is the almost complete exhaustion of the threonine pool (0.67 mM) from the culture medium. This confirms a previous study where threonine was, amongst other metabolites, taken up by *E. granulosus* metacestodes^40^, but this effect was not further investigated. The mucin-type glycoprotein Em2, synthesized in germinal layer cells and a major antigen of the laminated layer, is known to be rich in threonine and proline^70^. However, we could not detect any overuse of threonine for protein synthesis. Therefore, Em2 production cannot be the main cause of the high consumption of threonine by *E. multilocularis* metacestodes. Interestingly, threonine is known to be the only amino acid absolutely essential for the rapid division of mouse embryonic stem cells^71^. Threonine is hereby converted by threonine dehydrogenase and 2-amino-3-ketobutyrate coenzyme A ligase into glycine and acetyl-CoA, two metabolites that are then further used for purine synthesis and DNA-replication, and energy generation, respectively^71^. In the procyclic (insect stage) form of African trypanosomes, threonine is also converted via threonine dehydrogenase and 2-amino-3-ketobutyrate coenzyme A ligase, but finally feeding into fatty acid synthesis^72–74^. Whether the respective pathways are present and active in *E. multilocularis* should be further investigated. The release of glycine within our *in vitro* setup is in favor of according pathways being active. Other consumed amino acids, such as isoleucine, leucine, methionine, and valine, could also be metabolized into mitochondrial energy-generating pathways (Fig. 6). Glutamine was not detected by the methods used here, because the media used contained an altered, stabilized form of glutamine.

Taken together, this is the first characterization of the *in vitro* metabolomic footprint of *E. multilocularis* metacestodes and the respective composition of VF. The predominance of malate dismutation and the consumption of the amino acid threonine in *E. multilocularis* metacestodes *in vitro* lay the basis for further studies on potentially targetable pathways for targeting this most deadly of all helminth diseases.

## Supplementary information

Supplementary information

Supplementary information 1

Supplementary information 2
