# Author Correction: *In vitro* metabolomic footprint of the *Echinococcus multilocularis* metacestode

**DOI:** 10.1038/s41598-021-83843-4

**Published:** 2021-02-17

**Authors:** Dominic Ritler, Reto Rufener, Jia V. Li, Urs Kämpfer, Joachim Müller, Claudia Bühr, Stefan Schürch, Britta Lundström-Stadelmann

**Affiliations:** 1 Institute of Parasitology, Department of Infectious Disease and Pathobiology, Vetsuisse Bern, University of Bern, Bern, Switzerland; 2 Division of Systems and Digestive Medicine, Department of Surgery & Cancer, Imperial College London, London, United Kingdom; 3 Department of Chemistry and Biochemistry, University of Bern, Bern, Switzerland

Correction to: *Scientific Reports* 10.1038/s41598-019-56073-y, published online 19 December 2019

The Acknowledgements section in this Article is incomplete.

"We thank Caroline J. Sands (Imperial College London, UK) for the bioinformatic support of the NMR post processing pipeline. Many thanks to Andrew Hemphill (Institute of Parasitology, University of Bern) for helpful comments on the manuscript. The study was funded by the Swiss National Science Foundation (SNSF, 179439). D.R. was a recipient of the Karl Enigk Stiftung, Hannover, Germany, for parts of the project."

should read:

"We thank Caroline J. Sands (Imperial College London, UK) for the bioinformatic support of the NMR post processing pipeline. Many thanks to Andrew Hemphill (Institute of Parasitology, University of Bern) for helpful comments on the manuscript. The study was funded by the Swiss National Science Foundation (SNSF, 179439). D.R. was a recipient of the Karl Enigk Stiftung, Hannover, Germany, for parts of the project. JVL is funded by MRC New Investigator Grant (MR/P002536/1) and ERC Starting Grant (715662)."

