# Supplementary material for: In vitro metabolomic footprint of the Echinococcus multilocularis metacestode
Source: Sci Rep. 2019 Dec 19;9:19438. doi: 10.1038/s41598-019-56073-y (PMC6923418; doi:10.1038/s41598-019-56073-y)
Supplement: Supplementary file 1 — Supplementary information [file 41598_2019_56073_MOESM1_ESM.docx]

**Supplementary material for**

***In vitro* metabolomic footprint of the *Echinococcus multilocularis* metacestode**

**Dominic Ritler^1^, Reto Rufener^1^, Jia V. Li^2^, Urs Kämpfer^3^, Joachim Müller^1^, Claudia Bühr^3^, Stefan Schürch^3^, Britta Lundström-Stadelmann^1^**

**^1^Institute of Parasitology, Department of Infectious Disease and Pathobiology, Vetsuisse Bern, University of Bern, Switzerland**

**^2^Division of Systems and Digestive Medicine, Department of Surgery & Cancer, Imperial College London, United Kingdom**

**^3^Department of Chemistry and Biochemistry, University of Bern, Switzerland**

**Corresponding Author:**

**Britta Lundström-Stadelmann**

**Länggassstrasse 122**

**3012 Bern**

**Switzerland**

Britta.lundstroem@vetsuisse.unibe.ch

**Supplementary method 1. NMR sample preparation and data acquisition.**

400 µl of each sample were mixed with 250 µl sodium phosphate buffer (D_2_O:H_2_O, v/v, 9:1; pH = 7.4; 0.01% trimethylsilylpropanoic acid (TSP); 3 mM sodium-azide), vortexed and centrifuged. 600 µl of each sample were transferred into a 5 mm diameter NMR tube. ^1^H NMR spectroscopy was recorded at 300 K on a Bruker DRX 600MHz NMR spectrometer (Bruker Biospin; Rheinstetten, Germany) at 600.29 MHz. Each spectrum was acquired with 2.73 sec acquisition time for 128 scans (spectral width = 20.2 ppm) with a standard pulse sequence (recycle delay-90°−t_1_-90°-t_m_-90°-acquire free induction decay (FID)). Two-dimensional (2D) ^1^H-^1^H correlation spectroscopy (COSY),^1^H-^1^H total correlation spectroscopy (TOCSY), and ^1^H-^13^C heteronuclear single quantum coherence (HSQC) NMR were acquired from selected samples from each sample group (32 scans) to aid metabolite identification. This data is deposited freely accessible at <https://www.eudat.eu/>
(PID: 11304/0e960524-02dc-4bbb-ab26-792483388986,
DOI: 10.23728/b2share.cf3ac1ee15a743df99f044befea73f68) including all necessary metadata. NMR spectra were pre-processed by manual phasing and baseline correction. ^1^H NMR spectra were referenced to the TSP resonance at δ 0.0 for metabolite identification in TopSpin 3.0 (Bruker).

**Supplementary method 2. Preparation of germinal layer cells for amino acid quantification.**

To determine the amino acid content of germinal layer cells, 5 ml metacestode vesicles were incubated during 72 h in culture medium as described in method section 4, washed twice with 0.9 % NaCl, crushed with a 1 ml pipette and centrifuged at 300 x g for one minute at room temperature. The pellet containing laminated and germinal layers was washed 4 times with 0.9 % NaCl and resuspended in 1 ml H_2_O. For lysis of germinal layer cells, the material was freeze-thawed on dry ice four times. The cell lysate was centrifuged at 9’300 x g for 10 min, 4°C and 10 µl supernatant was either analyzed directly or hydrolyzed (for analysis of proteome composition) according to Chang and Knecht^75^. Amino acid analysis was performed as described in **supplementary methods 3**.

### Supplementary method 3. Amino acid quantification by HPLC.

High-performance liquid chromatography (HPLC) was performed as described^76^ to quantify amino acids in various samples (vcDMEM, ccDMEM, VF, germinal layer lysates, germinal layer proteome). Phenylthiocarbamyl (PTC)-amino acids were analyzed by RP-HPLC on a Nova Pack C18 (4 µm, 3.9 x 150 mm) column (Waters, Baden, Switzerland) using a Dionex Summit HPLC (Dionex, Olten, Switzerland). The original Buffer A was replaced by 0.14 M ammonium acetate. Buffer B was 60 % acetonitrile in water. The Pierce amino acid standard H (Thermo Scientific, Reinach, Switzerland) (2.5 mmol/ml) was used for calibration. The standard solution was diluted 1:20 and 20 µl were dried and reacted with the Edman reagent. 20 % of the derivatized material (corresponding to 500 pmol) was analyzed by HPLC. The flow rate was 1 ml/min and an optimized gradient (0 - 3 min 3 % B, 3 - 13 min: 3 - 46 B) was applied. Quantitation was based on the peak heights.

**Supplementary table 1. Metabolite peak intensities in VF from *in vitro* grown *E. multilocularis* metacestodes.** Given are metabolite groups, metabolite names, the median integral (n = 5) with the median absolute deviation (MAD) in parentheses. Asterisks indicate not detectable peaks (baseline levels).

| Metabolite  group | Metabolite name | Median integral (MAD) for VF |
| --- | --- | --- |
| Amino acids | Alanine | 292.36 (±13.93) |
|  | Aspartate | * |
|  | Glycine | 98.01 (±1.57) |
|  | Isoleucine | 71.14 (±0.54) |
|  | Leucine | 117.71 (±1.20) |
|  | Lysine | 99.90 (±2.89) |
|  | Methionine | 16.49 (±0.35) |
|  | Phenylalanine | 22.67 (±0.34) |
|  | Threonine | * |
|  | Tryptophan | 2.99 (±0.09) |
|  | Tyrosine | 29.69 (±0.39) |
|  | Valine | 102.11 (±0.95) |
| Organic acids | Acetate | 378.65 (±35.69) |
|  | Formate | 3.00 (±0.23) |
|  | Fumarate | 4.93 (±0.34) |
|  | Lactate | 116.73 (±6.66) |
|  | Malate | 28.69 (±1.30) |
|  | Succinate | 1269.41 (±131.10) |
| Sugars | Glucose | 473.56 (±9.85) |
|  | Myo-inositol | 50.41 (±0.30) |
| Organic compounds | Acetone | 2.64 (±0.05) |
|  | Ethanol | 35.16 (±0.54) |

**Supplementary figure 1. 1D ^1^H NMR spectra of host cell preconditioned culture media incubated with *E. multilocularis* metacestodes (vcDMEM), corresponding control media (ccDMEM), and *E. multilocularis* vesicle fluid (VF) extracted from metacestodes.** a) to c) representative NMR spectra (600 MHz) of *E. multilocularis* metacestode-incubated media a) vcDMEM, b) ccDMEM controls, and c) vesicle fluid (VF). Annotations for amino acids are given in the three-letter format (shown in b), all other metabolites are labeled in a) and c). The x-axis depicts the chemical shifts in ppm. TSP was used as chemical shift reference (*δ* 0.0 ppm). Note the strong changes between vcDMEM and ccDMEM in the levels of the metabolites acetate, alanine, aspartate, fumarate, glucose, malate, succinate, and threonine. TSP = trimethylsilylpropanoic acid.

**Supplementary figure 2. Median frequencies of amino acids in *E. multilocularis* germinal layer cell proteins.** Frequencies were determined by HPLC (n = 3, error bars = range).

**References**

75. Chang, J. Y. & Knecht, R. Direct analysis of the disulfide content of proteins: methods for monitoring the stability and refolding process of cystine-containing proteins. *Anal. Biochem.* **197**, 52–58 (1991).

76. Bidlingmeyer, B. A., Cohen, S. A. & Tarvin, T. L. Rapid analysis of amino acids using pre-column derivatization. *J. Chromatogr. B. Biomed. Sci. App.* **336**, 93–104 (1984).
